# No Effects of Anodal tDCS on Local GABA and Glx Levels in the Left Posterior Superior Temporal Gyrus

**DOI:** 10.3389/fneur.2018.01145

**Published:** 2019-01-08

**Authors:** Gerard E. Dwyer, Alexander R. Craven, Marco Hirnstein, Kristiina Kompus, Jörg Assmus, Lars Ersland, Kenneth Hugdahl, Renate Grüner

**Affiliations:** ^1^Department of Biological and Medical Psychology, University of Bergen, Bergen, Norway; ^2^NORMENT Centre of Excellence, Haukeland University Hospital, Bergen, Norway; ^3^Centre for Clinical Research, Haukeland University Hospital, Bergen, Norway; ^4^Department of Clinical Engineering, Haukeland University Hospital, Bergen, Norway; ^5^Division of Psychiatry, Department of Clinical Medicine, Haukeland University Hospital, Bergen, Norway; ^6^Department of Radiology, Haukeland University Hospital, Bergen, Norway; ^7^Department of Physics and Technology, University of Bergen, Bergen, Norway

**Keywords:** tDCS, GABA, Glultamate, magnetic resonance spectroscopy, MRS

## Abstract

A number of studies investigating the biological effects of transcranial direct current stimulation (tDCS) using magnetic resonance spectroscopy (MRS) have found that it may affect local levels of γ-aminobutyric acid (GABA), glutamate and glutamine (commonly measured together as “Glx” in spectroscopy), and N-acetyl aspartate (NAA), however, these effects depend largely on the stimulation parameters used and the cortical area targeted. Given that different cortical areas may respond to stimulation in different ways, the purpose of this experiment was to assess the as yet unexplored biological effects of tDCS in the posterior superior temporal gyrus (pSTG), an area that has attracted some attention as a potential target for the treatment of auditory verbal hallucinations in schizophrenia patients. Biochemical changes were monitored using continuous, online MRS at a field strength of 3 Tesla. Performing intrascanner stimulation, with continuous spectroscopy before, during and after stimulation, permitted the assessment of acute effects of tDCS that would otherwise be lost when simply comparing pre- and post-stimulation differences. Twenty healthy participants underwent a repeated-measures experiment in which they received both active anodal and sham intrascanner stimulation in a stratified, randomized, double-blind experiment. No significant changes in GABA, Glx, or NAA levels were observed as a result of anodal stimulation, or between active and sham stimulation, suggesting that a single session of anodal tDCS to the pSTG may be less effective than in other cortical areas that have been similarly investigated.

## Introduction

Transcranial direct current stimulation (tDCS) is a non-invasive neurostimulation technique that uses constant, low level (0.5–2.0 mA) direct current to modulate cortical excitability in a polarity dependent manner (1). Nitsche and Paulus (2) used the magnitude of motor-evoked potentials (MEP) as generated by transcranial magnetic stimulation (TMS) as an indication of changes in excitability and found that tDCS was able to induce changes in excitability of up to 40%, with anodal stimulation having an excitatory effect, and cathodal stimulation having an inhibitory effect. Subsequent studies showed that effects may outlast the duration of stimulation, with short applications inducing excitability shifts during stimulation, and ~10 min or more of stimulation producing persistant effects lasting up to 90 min after current flow has ceased (3) suggesting that tDCS has the ability to induce long term potentiation (LTP)-like effects on synaptic plasticity (4).

Due to its purported effects on excitability and synaptic plasticity, tDCS has been investigated as a potential treatment for a range of neurological and psychiatric disorders such as Parkinson's disease (5), depression (6), and for the treatment of auditory verbal hallucinations in schziophrenia. A case reported by Homan et al. (7) found that cathodal tDCS halfway between T3 and P3 in the 10–20 electroencephelography (EEG) system was successful in alleviating both hallucinations (−60% Hallucination Change Scale (HCS) score) and global symptoms (−20% Postive and Negative Syndrome Scale (PANSS) score). A randomized control trial conducted by Brunelin et al. (8) using a similar stimulation paradigm at 2.0 mA also showed improvement in hallucinations (−31% Auditory Hallucination Rating Scale (AHRS) score) and global symptoms (−13% PANSS score). Subsequent studies, using similar stimulation parameters have found both reductions (9) and no significant differences (10, 11) in symptoms. While tDCS shows great promise as a potential treatment for schizophrenia, the lack of consistent findings between these studies highlight the need for a deeper understanding of the effects of tDCS.

Although generally accepted that anodal stimulation typically facilitates excitability and cathodal stimulation inhibits excitability (12), studies have shown that the effects of tDCS on excitability are not so simplistic, and depend on a number of factors such as electrode size and placement, stimulation intensity and duration, as well as the orientation of neurons relative to the stimulating electrodes (12–14). Furthermore, Batsikadze et al. (15) found that while 20 min of cathodal stimulation at 1.0 mA had an inhibitory effect, 20 min of cathodal stimulation at 2.0 mA had an excitatory effect, increasing the magnitude of measured MEPs. Esmaeilpour et al. (16) showed that the dose-response relationship in tDCS is not necessarily linear, and that although increasing current produces a corresponding increase in brain electric field, it may not necessarily enhance a neurophysiological, behavioral or clinical outcome. As Woods et al. (14) caution, it cannot be taken for granted that what is effective in a particular cortical area is transferable and applicable to others, rather recommending a “titration” of parameters.

Despite the observed effectiveness of tDCS, the exact mechanisms by which it works are not yet fully understood. Horvath et al. (17) show that changes in cognitive effects alone may be an unreliable measure of effectiveness. Computational forward models and simulations have been useful in imaging current flow, aiding in the design of stimulation paradigms (18) but do not provide information about neuronal responses to delivered current or whether the effect is excitatory or inhibitory in nature.

Krause et al. (19) suggest that tDCS may modulate the excitation/inhibition balance, that is, the relative contributions of excitatory and inhibitory inputs to a neural circuit corresponding to a neuronal event. Using *in vivo* magnetic resonance spectroscopy (MRS), the excitation-inhibition balance may be characterized in terms of the local concentrations of the excitatory neurotransmitter glutamate and inhibitory neurotransmitter gamma-aminobutyric acid (GABA). Studies that have used MRS to investigate the effect of tDCS have found anodal tDCS to reduce local cortical GABA concentration in the motor cortex (20, 21) and to increase local concentrations of glutamate and glutamine, measured together as “Glx,” and N-acetyl aspartate (NAA) in the intraparietal and prefrontal cortices (22, 23), the observed reduction in inhibitory neurotransmitter levels and concurrent increases in excitatory neurotransmitter levels being consistent with the facilitatory nature of anodal stimulation. Thus, *in vivo* MRS provides a window into the biochemical events underlying tDCS that may also be used as a biomarker indicating the effectiveness and nature of a stimulation paradigm.

In this study, MRS was used to investigate the acute biochemical effects of tDCS in validating its potential for use as a treatment for auditory-verbal hallucinations in schizophrenia. However, rather than simply comparing pre- and post-stimulation spectral acquisitions, biochemical changes were measured continuously using online MRS in a manner similar to those used by Bachtiar et al. (24) and Hone-Blanchet et al. (23). By acquiring spectra continuously over the course of stimulation, spectral frames could be combined in such a way that metabolite levels could be measured and tracked before, during and post stimulation, allowing better insight into the acute effects of stimulation as opposed to the lasting effects. Findings in other cortical areas suggest that if anodal tDCS were to have a similar effect on the local excitation-inhibition balance, it may be measured as a statistically significant increase in Glx and NAA levels (22, 25) and decrease in GABA levels (20, 21, 24) and that these changes would be significantly different under active stimulation when compared to sham.

## Materials and Methods

This study was carried out in accordance with the recommendations and ethical approval of the regional committee for medical and health research ethics (REK-Vest) REK case number 2013/2342. All subjects gave written informed consent in accordance with the Declaration of Helsinki and the guidelines drawn up by The Norwegian National Research Ethics Committee for medical and health research (NEM).

### Participants

Twenty healthy participants (mean age: 25 years, range: 19–32; 10 male) participated in the study. All participants were required to complete a Norwegian language version of the Edinburgh handedness inventory (26) to determine right-handedness in an attempt to control for issues related to lateralization of cortical areas, such that stimulation in the left hemisphere affects approximately the same functional area in each participant. The test assessed dominance of right and left hand in performing 10 everyday activities to produce a score ranging between −100 (exclusively left handed) and +100 (exclusively right handed), participants with a score greater than +40 were considered to be right handed and were permitted into the study (mean score: +80, SD: 24). Based on self-report, participants were free from psychiatric and neurologic conditions and had not used any psychoactive/psychotropic substances, including no smoking or other tobacco based or nicotine containing products, for 6 months prior to participating in the experiment. Participants were also instructed not to consume alcohol for at least 24 h prior to participation.

Data from one female participant was omitted from final analyses due to abnormally high measurements of Glx more than three standard deviations above the group mean (Glx levels almost 5 times higher than average values), suggesting an error in spectral acquisition.

### tDCS Stimulation

Stimulation was performed using an MR-compatible DC-Stimulator MR (neuroConn GmbH, Ilmenau, Germany) fitted with two 5 × 7 cm (35 cm^2^) MR compatible rubber electrodes. Given that the motivation for this study was the potential for tDCS to be used as a treatment for schizophrenia, stimulation parameters were chosen to emulate those used in previous studies. Intensity was set at 2.0 mA (27) and although the majority of studies using tDCS as a treatment for auditory-verbal hallucinations have stimulated for 20 min, the prohibitively long scan time this would necessitate in order to have three equally long spectroscopy windows meant that stimulation had to be limited to 10 min. The anodal electrode was placed with the center of the pad on an area over the pSTG, such that the lower corners of the 7 cm edge of the electrode touch points T3 and T5 in the EEG 10-20 system. The cathodal electrode was placed over the contralateral orbitofrontal cortex, a site commonly used in tDCS montages for placement of the reference electrode (2, 12) such that the center of the electrode covered point AF8 in the EEG 10–20 system. Each electrode was coated with a layer of Ten20 conductive paste (Weaver and Company, Aurora, United States of America) at the interface between electrode and skin to improve both adhesion and conductivity. Once the electrodes were in place, participants were placed in the scanner with electrodes attached but not connected to the stimulation box. Electrodes were only connected prior to spectroscopy sequences.

This study followed a stratified, randomized, double-blind design, with both participants and experimenters blind to the stimulation condition. Each subject participated in two MR-scanning sessions with tDCS: one with active and one with sham stimulation, separated by a wash-out period of 1 h outside of the scanner (12, 28) counterbalanced for order. Double-blinding was performed by having the stimulation condition determined by a code, independently predetermined by a researcher not present at the stimulation, such that each participant underwent both active and sham stimulation conditions and that equal numbers experienced active and sham stimulation as the first condition.

### MR-Imaging and Spectroscopy

All imaging and spectroscopy was performed on a 3 T GE 750 Discovery Scanner from GE Healthcare (General Electric, Milwaukee, United States of America) using a standard 8-channel head coil from Invivo (Invivo corp., Gainsville, Florida, United States of America).

**Figure 1:**
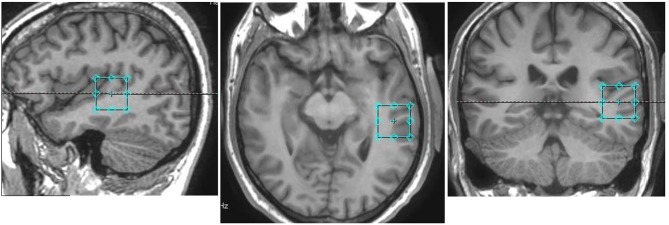
Voxel placement in the pSTG in one participant: Sagittal (left), axial (middle), and coronal (right) views overlayed on an anatomical scan.

Following a 3-plane localizer sequence (2D Spin Echo, TE = 80 ms, FOV = 240 mm, slice thickness = 8 mm, slice spacing = 15 mm) structural anatomical imaging was performed using a 3D T1 weighted fast spoiled gradient sequence (FSPGR) (number of slices = 192, slice thickness = 1.0 mm, repetition time (TR) = 7.8 ms, echo time (TE) = 2.95 ms, field of view = 260 × 260 mm^2^, flip angle = 14 degrees, matrix = 256 × 256). These structural images were used to position a 24 × 24 × 24 mm^3^ voxel for the spectroscopy component of this experiment in the left pSTG, centered around the primary auditory cortex, aligned orthogonally in the axial scan plane with no angulation (Figure 1).

**Figure 2:**
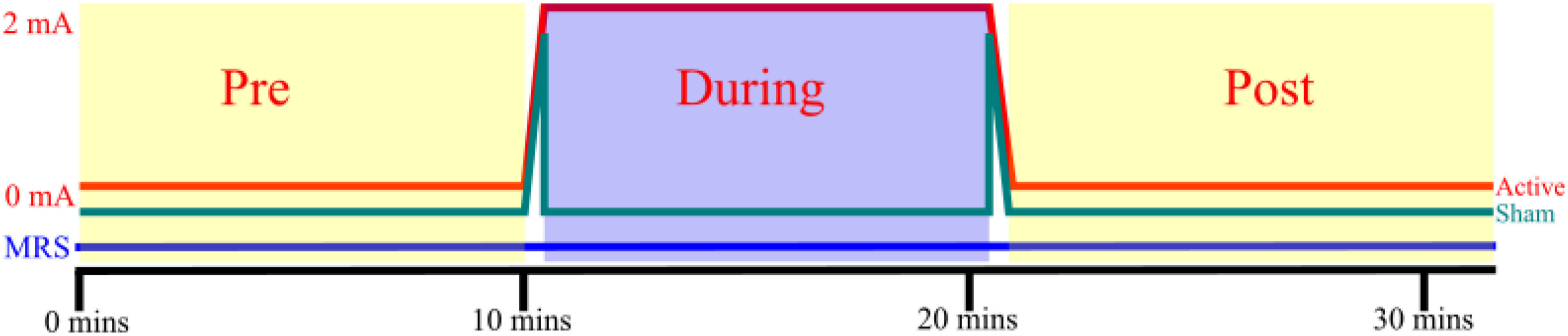
tDCS and MRS: Each participant received both active and sham stimulation, separated by a washout period of 1 h, counterbalanced for order. 24 s of ramping up and down were incorporated into both active and sham stimulation. MRS was acquired constantly throughout each session. The pre-, during, and post-stimulation spectroscopy windows did not include frames acquired during ramping.

Since the aim of this study was to characterize acute biochemical changes in terms of the excitation-inhibition balance, a GABA specific MEGA-PRESS sequence (29) was used as it provides accurate and stable measurements of GABA, as well as a measurement of glutamate and glutamine combined as “Glx” (30). Spectroscopy was performed using a MEGA-PRESS sequence (TE = 68 ms, TR = 1,500 ms, 8-way phase cycling, editing at 1.9 and 7.5 ppm in alternating frames) of 628 paired repetitions, followed by 16 unsuppressed reference acquisitions for a total scan time of 31 min and 48 s. Once 10 min of spectroscopy had elapsed, stimulation was initiated at the control box located outside the scanner at the control room. Active stimulation was delivered for 10 min with 24 s of ramping time both before and after the stimulation/sham period at a constant intensity of 2.0 mA. For the sham stimulation condition, intensity was ramped up to 2.0 mA over 24 s, then delivered for another 40 s, before being ramped down to zero, giving participants a similar sensation to that they would experience during active stimulation. Spectroscopy acquisition continued for 10 min in order to assess post-stimulation effects (Figure 2).

### Spectral Analysis

While no spectral artifacts were observed during steady-state tDCS stimulation, mild artifacts were seen in spectral frames acquired during the ramping periods for both active and sham stimulation. Frames from these periods were omitted from all subsequent analyses.

Following phase adjustment, coil combination, and realignment, each continuous acquisition was first subdivided into three smaller blocks of ~10 min, with exact length depending on how many frames were excluded due to ramping artifacts, comprising a pre-, during-, and post stimulation block for each session, hereafter referred to as a three point analysis. Frames within each block were then averaged together and within each block, ON, and OFF spectrum pairs were subtracted to produce a difference spectrum then subjected to quantitative analysis with LCModel (version 6.3-1J) (31, 32) using a simulated basis set (33) with Kaiser coupling constants (34) to provide an estimate of average levels of GABA, glutamate and glutamine measured together as Glx, glutathione (GSH), NAA, and N-acetyl aspartate glutamate (NAAG). Metabolite levels were scaled relative to the unsuppressed water signal acquired at the end of each spectroscopy sequence.

One issue that affects MEGA-edited GABA spectroscopy is co-editing of macromolecule (MM) resonances at 1.7 ppm contaminating the GABA signal in the difference spectrum. GABA, in this report, refers to both GABA and the co-edited macromolecule, typically denoted GABA+ (35).

To further investigate acute effects of tDCS, and eliminate the possibility short-lived metabolic fluctuations being obscured through averaging, a second analysis was performed in which the during- and post-stimulation blocks were further subdivided into two smaller windows in an attempt to uncover any changes in metabolite concentration during this period, thus providing five time points over the acquisition, hereafter referred to as the five point analysis: one 10 min pre-stimulation window, two 5 min during-, and two 5 min post-stimulation windows.

MRS signals have been demonstrated to be susceptible to line-broadening artifacts associated with local blood-oxygen-level dependent (BOLD) effects (36). As an indication of potential BOLD interference, the full width at half maximum (FWHM) values as determined by LCModel were used as a measure of quality control, to ensure the MRS signal had not been significantly affected between time points.

### Statistical Analysis

Statistical analyses were performed using R (37) and the nlme package (38) to perform a linear mixed effects model analysis of the effect of tDCS on the concentrations of three metabolites of interest, namely NAA, Glx, and GABA, over time. This model specified two groups of participants (active-first and sham-first) and time period as fixed effects as well as an interaction effect between the two, with the subject as a random effect. This model was also used to investigate crossover effects between the active and sham stimulation conditions due to the within-subject design of the study, to determine whether order of stimulation, active first or sham first, may have had any significant effect on results and whether the stimulation condition in the first session had any lasting effect on the second. The same model was used for both the 3-point and 5-point analyses.

## Results

**Figure 3:**
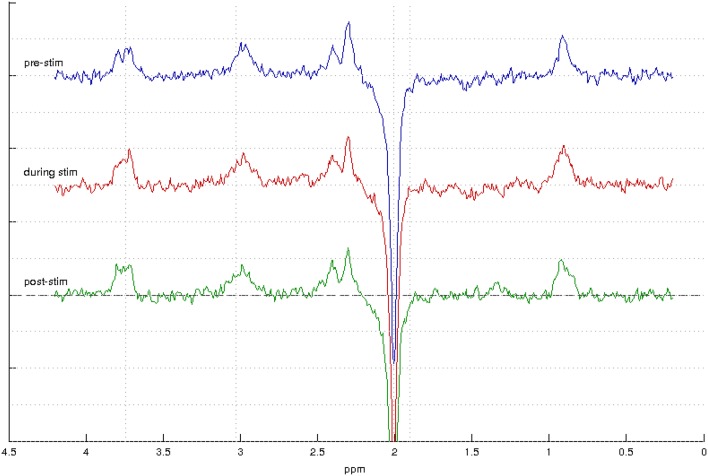
MEGA-PRESS spectra from one participant showing spectra acquired during the pre-stimulation window (top, blue) during stimulation (middle, red) and post-stimulation (bottom, green).

**Figure 4:**
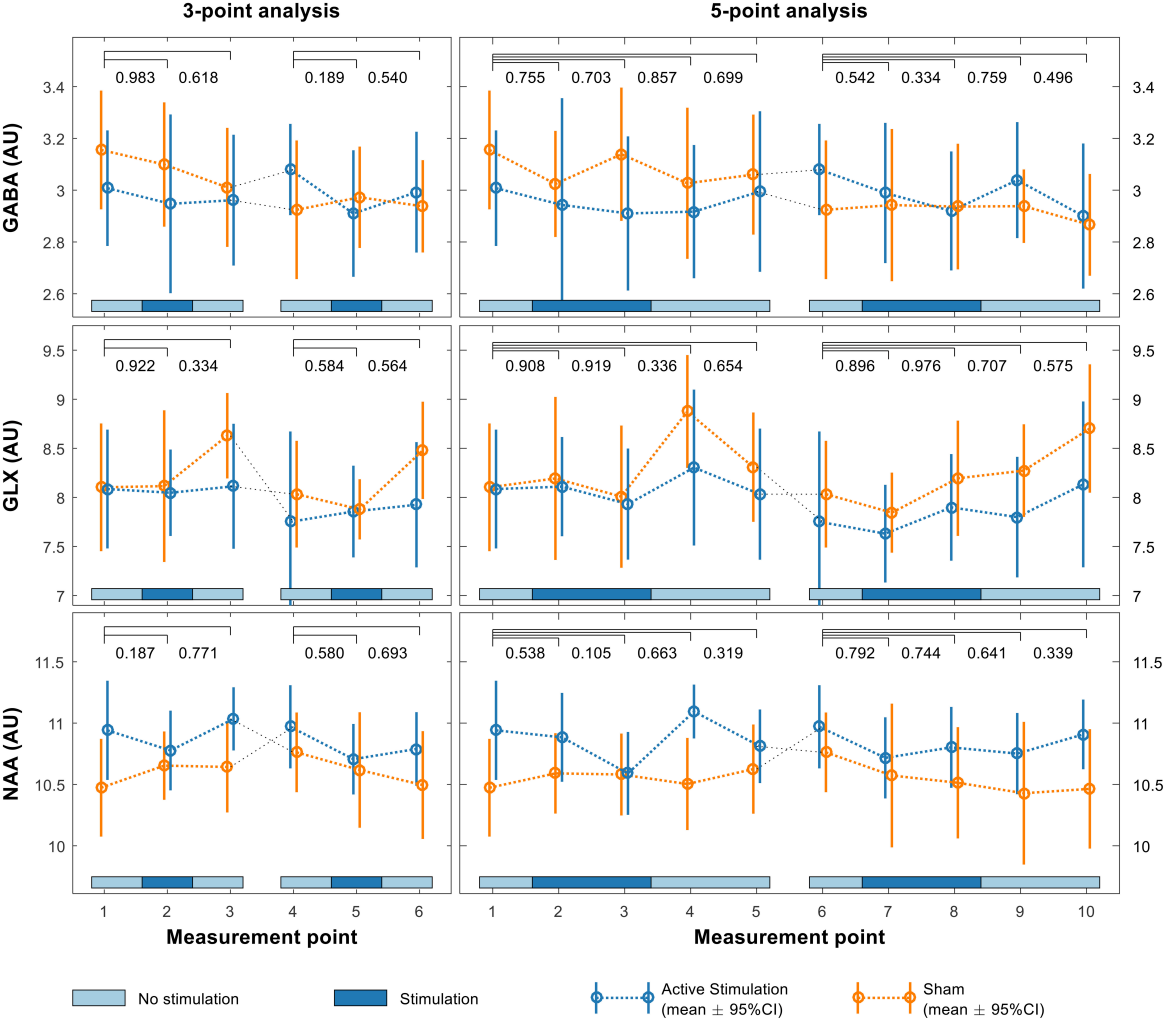
Linear mixed effects model of GABA (upper row), Glx (middle row) and NAA (lower row) levels in the pSTG for both the first and second sessions using both 3-point (left) and 5-point (right) analyses. *p*-values shown above each plot indicate significance of interaction effects at each time point relative to baseline.

Sample spectra from the three-point analysis of an individual participant are shown in Figure 3 along with spectral quality metrics for all participants in Table 1. A linear mixed effects model of the average metabolite concentration across three time windows (pre-, during-, and post-stimulation) revealed no significant fluctuations in any of the metabolites of interest between any time points (Figure 4 and Appendix A). Similarly, no significant fluctuations in any of the metabolites of interest were found between any time points in the five-time point analysis (Figure 4 and Appendix B).

**Table 1:** Spectral Quality: FWHM, SNR, and mean %CRLB for GABA and Glx for each stimulation window.

**Window**	**FWHM (Hz)**	**SNR**	**Mean GABA**	**Mean Glx**
			**%CRLB**	**%CRLB**
Pre Stim	8.22 ± 2.34	20.47 ± 4.71	5.12	4.54
During Stim	8.39 ± 2.41	20.83 ± 4.20	5.16	4.41
Post Stim	8.03 ± 2.18	21.67 ± 4.04	4.91	4.24

No significant crossover effects were found (Figure 4, Appendices A, B) indicating both that the order in which participants received the two different stimulation conditions had no significant effect on results and that there were no crossover effects from the first session significantly affecting the second. There was no significant difference in the change between groups over time, indicating no difference in fluctuations for any of the metabolite levels between active and sham conditions.

The FWHM as reported by LCModel was used as an indication of potential BOLD interference (Tables 2, 3), but saw very little fluctuation between time points, making BOLD interference an unlikely source of error.

**Table 2:** Average FWHM and standard deviation (sd) as estimated by LCModel for the 3-point analysis.

**Mean FWHM−3-point analysis (Hz)**						
	**Pre**	**sd**	**During**	**sd**	**Post**	**sd**
Active	7.95	1.86	7.98	1.79	7.59	1.38
Sham	8.46	2.24	8.64	2.24	8.32	2.30

**Table 3:** Average FWHM and standard deviation (sd) as estimated by LCModel for the 5-point analysis.

**Mean FWHM−5-point analysis (Hz)**										
	**Pre**	**sd**	**During1**	**sd**	**During2**	**sd**	**Post1**	**Sd**	**Post2**	**sd**
Active	7.95	1.86	8.00	1.77	7.94	1.69	7.74	1.73	7.77	1.55
Sham	8.46	2.24	8.50	0.02	8.46	2.06	8.28	2.42	8.46	1.96

## Discussion

The montage and stimulation parameters used in this experiment did not induce a statistically significant effect on Glx, GABA, or NAA levels as measured with the MRS sequence used, and there was no significant difference in response observed between the active and sham stimulation conditions.

The active hypothesis for this experiment was informed by previous studies in which active anodal stimulation was found to be associated with increases in Glx and NAA levels (22, 23) and decreases in GABA levels (20, 21, 24) as measured by MRS. In comparing these studies with the findings presented here, there are three key elements to be considered, namely the stimulation parameters, the MRS acquisition parameters and the site of stimulation and spectroscopy.

As stated in section tDCS Stimulation, due to limitations of the experimental design, stimulation could only be delivered for 10 min as opposed to the 20 min previously used in the treatment of schizophrenia symptoms. Although as little as 7 min of stimulation has been shown to induce lasting effects after stimulation has ceased (39), it cannot be taken for granted that the 10 min delivered in this session was sufficient to induce a change. While the stimulation window was shorter than the 30 min used by both Clark et al. (22) and Hone-Blanchet et al. (23) and the 20 min and 15 min used by Bachtiar et al. (24) and Kim et al. (20), respectively, Stagg et al. (21) were able to detect significant changes in GABA and Glx levels in the left sensorimotor cortex using a similar MEGA-PRESS sequence at 3 T given only 10 min of anodal stimulation at 1.0 mA. The findings of Batsikadze et al. (15) and Esmaeilpour et al. (16) suggest it is possible that stimulating at 2.0 mA had a different effect to the one predicted. However, studies conducted by Brunelin et al. (8) and Mondino et al. (9) both found significant reductions in symptoms of auditory-verbal hallucinations using stimulation in this area following cathodal stimulation at 2.0 mA, suggesting an issue more likely related to electrode polarity than stimulation intensity. While no significant changes, nor non-significant tendencies toward changes in any of the metabolites under investigation were seen during stimulation, even in the five-point analysis, it is unlikely that allowing a full 20 min of stimulation would induce a measureable effect, though it cannot be ruled out conclusively.

One of the unique features of this study was the use of continuous, online MRS as opposed to separate acquisitions. While Hone-Blanchet et al. (23) also acquired spectra during stimulation, also using a MEGA-PRESS sequence with an echo time of 68 ms and 11 min acquisition blocks, their study does not include a pre-stimulation window. Similarly, Clark et al. (22) acquired multiple spectra during the pre- and post-stimulation windows, also using a MEGA-PRESS sequence with an echo time of 68 ms, but with spectra acquired sequentially rather than continuously in blocks of 4 min and 48 s. While there is little difference in terms of the resultant spectra whether acquired continuously or sequentially, acquiring separate scans may introduce more variability as each pre-scan affects parameters such as shim, gain adjustment and center-frequency tuning between each segment. It may be considered more robust to acquire all spectra with the same parameters, as was done in this study with single continuous acquisitions. Compared with previous studies using similar sequences, comparable or shorter acquisition times, and smaller voxel sizes, i.e., 20 × 20 × 20 mm^3^ (21, 22, 24), there is little evidence to suggest an error in the MRS acquisition. Intuitively, a larger voxel size provides a higher signal-to-noise ratio, but may come at the expense of some focality in terms of covering the site of stimulation. It is possible that the larger voxel size used in this study may have incorporated spectra from cells not affected by stimulation. However, the voxel dimensions are still small compared to the surface area of the stimulating electrode, and tDCS is not a particularly focused stimulation technique.

The most significant difference between this study and other studies that have measured biochemical changes associated with tDCS with MRS is the cortical region being investigated, both as a stimulation site and volume of interest in spectroscopy. As Woods et al. (14) illustrated, it cannot be taken for granted that all cortical areas will respond to stimulation in the same manner, and compared to areas such as the sensorimotor cortex and frontal areas such as the dorsolateral prefrontal cortex, the temporoparietal and temporal regions have not been quite as thoroughly investigated. One study investigating the use of anodal tDCS in an adjacent cortical area, namely the left mid-posterior temporal gyrus, on improving performance in a range or reading and naming tasks (40) did not find any significant improvement in performance. Although different stimulation parameters were used, the agreement between the null-findings of this and the present study suggest it is possible that the pSTG and adjacent areas in the region, are not as responsive to anodal stimulation as other areas that have been investigated, but that the effectiveness of tDCS as a treatment for hallucinations is based on its ability to modulate over-active areas in the brain with cathodal stimulation. That is to say, anodal stimulation may not affect excitability in the pSTG, but cathodal stimulation may be effective in modulating activity in over-active or pathologically active networks such as those that might be associated with hallucinations. Computer modeling may be able to determine whether the responsiveness of this cortical area may be due to anatomical features such as skull thickness or cerebrospinal fluid density. It may be of interest to repeat a similar experiment looking at the effects of cathodal stimulation in this area in conjunction with computer models that may be able to determine whether the absence of an observed affect may be attributed to issues of anatomy and current flow.

In an investigation into the effect of active, intrascanner tDCS on the BOLD response as measured with functional MRI, Antal et al. (41) found that the presence of an electric current in the magnetic field inside an MRI scanner produces artifacts that may result in confounding false-positive activity patterns. While mild artifacts were observed during the ramping periods before and after stimulation, and these spectral frames were removed from subsequent analyses, there were no artifacts observed during active or sham stimulation periods. Furthermore, there were no statistically significant differences observed between the pre- and post-stimulation windows, where no ongoing active or sham stimulation was present. This, coupled with the findings of previous studies using online MRS acquired during stimulation (23, 24) suggest that interfence caused by ongoing intrascanner tDCS during spectral acquisition is not a likely source of error.

Another potential explanation for the null findings of this experiment is insufficient power as a result of too few participants. An analysis conducted in G^*^Power (42) determined there were enough participants to detect at least a medium sized effect (i.e., effect size > 0.6, 1-β = 0.8, α = 0.05). Many of the studies that have previously investigated biochemical effects of tDCS have noted significant findings with smaller sample sizes than the 19 used in this study, including *N* = 12 (22), *N* = 17 (23), and *N* = 11 (21). To this end it is believed that the study was sufficiently powered, in terms of the participant sample size, to detect a comparable effect. One of the problems with statistical power as outlined by Button et al. (43) is that while problems of low statistical power are typically associated with reduced chances of detecting a true effect, they may also reduce the likelihood of a statistically significant result being indicative of a true effect. That is, finding false positive effects due to inflated effect sizes. As Westwood et al. (40) illustrate, while it may be of value to include more participants in future studies, it calls the effectiveness of a single session of tDCS into question if the effects are so small. Referring to a meta-analysis in preparation, Westwood et al. (40) discuss an analysis of pooled studies looking at anodal stimulation in the frontal and temporal lobes which produced a sample size of almost 200 participants in which there was still no evidence of an effect of a single session of tDCS. In light of this, it is not believed that an increased sample size would have improved the outcome of this experiment.

One problem affecting the spectroscopy aspect of this study is that of how to quantify metabolite levels. Typical methods make use of water as an endogenous reference, or report the concentration as a ratio relative to an internal reference such as creatine or NAA. While creatine is typically favored as an internal reference (44) its use is complicated when using the MEGA-PRESS sequence as creatine signals are eliminated during subtraction and are not present in the difference edited spectrum, though they may be recovered from the spectra acquired without an editing pulse (commonly referred to as the “OFF” spectrum in the spectral pairs used to create the difference spectrum). NAA was not used as an internal reference as it has been demonstrated to be affected by anodal tDCS (22, 23), although no changes in NAA levels were measured over the course of the acquisition. The use of water as an endogenous reference can be problematic for studies such as this that attempt to measure metabolic changes in a dynamic manner, i.e., in relation to activity over time, as MRS signals have been shown to be susceptible to line-broadening artifacts associated with local BOLD effects (36). Using a fixed water reference taken at the end of the acquisition, as was done in this study, the reference signal was not subject to fluctuations as the result of a BOLD effect throughout the scan as the metabolites of interest were, i.e., comparing an unchanging reference to a signal subject to interference may increase the likelihood of a false change being detected. As a single, fixed water reference was used, it is difficult to decisively rule out any incidental BOLD-related fluctuations. However, such fluctuations would likely be manifest across all metabolites in the FWHM estimate given by LCModel, which is not seen in our data (Tables 2, 3), making it unlikely to be a significant source of error. Ideally, an experiment such as this would benefit from the use of external referencing, such as the Electronic Reference To access *in vivo* Concentrations (ERETIC) method (45, 46).

In interpreting these findings, it is important to consider that tDCS is regarded as a neuromodulatory technique, it does not induce activity or action potentials, but rather facilitates increases or decreases in neuronal excitability. Bikson and Rahman (47) discuss the idea of activity-selectivity and task-specific modulation, that is, that tDCS will preferentially modulate a neuronal network that is already active, while not modulating a separate network that is inactive. One of the problems with the region of interest in this study is that it contains the primary auditory cortex and adjacent areas responsible for the sensation of sound and processing of speech (48). While other paradigms have investigated cortical areas that may be associated with a task, e.g., the primary motor cortex and force adaptation task (20), that may distinguish between blocks of activity and rest, the auditory cortex will experience ongoing sensory input during scanning. It is possible that no biochemical changes were observed between blocks as the local cortical circuit was already in an active state during the pre-stimulation window and that tDCS was not able to drive a higher level of activity.

In conclusion, using continuous online MRS, no significant change in the levels of Glx, GABA, or NAA in the left pSTG was observed that could be attributed to an effect of active, anodal tDCS. Despite this, the method provides a useful insight into the acute effects of stimulation paradigms and their effect on local neuronal circuitry. Further research investigating an effect of tDCS in this area suggests performing a similar experiment using cathodal tDCS, redesigning the experiment to allow 20 min of stimulation, perhaps combining this experiment with computer models and also using an external referencing method to avoid possible confounding variables associated with how metabolite levels are measured.

## Author Contributions

GD, AC, MH, KK, KH, and RG were involved in the conception and design of the study. GD, AC, MH, and KK contributed to planning and performing of the experiments. AC performed the spectral analysis component of this study. JA performed statistical analyses. LE contributed to acquisition of MR-Spectra. GD wrote the manuscript with the assistance and critical feedback of all contributing authors.

### Conflict of Interest Statement

We wish to draw to the attention of the Editor that co-authors AC, LE, KH, and RG have shares in the company NordicNeuroLab A/S which produces add-on equipment for MRI examinations that were used in this study. The remaining authors declare that the research was conducted in the absence of any commercial or financial relationships that could be construed as a potential conflict of interest.

## Abbreviations

BOLD: Blood Oxygen Level Dependent
EEG: Electroencephalography
ERETIC: Electronic reference to access *in vivo* concentrations
FWHM: Full width at half maximum
GABA: Gamma-aminobutyric acid
Glx: Glutamate and glutamine
GSH: Glutathione
LTP: Long-term potentiation
MEGA-PRESS: Mescher-Garwood point resolved spectroscopy
MEP: Motor evoked potential
MRS: Magnetic Resonance Spectroscopy
NAA: N-acetylaspartate
NAAG: N-acetylaspartylglutamate
pSTG: Posterior superior temporal gyrus
tDCS: Transcranial direct current stimulation
TE: Echo time
TMS: Transcranial Magnetic Stimulation
TR: Repetition time
