# Supplementary material for: No Effects of Anodal tDCS on Local GABA and Glx Levels in the Left Posterior Superior Temporal Gyrus
Source: Front Neurol. 2019 Jan 8;9:1145. doi: 10.3389/fneur.2018.01145 (PMC6332511; doi:10.3389/fneur.2018.01145)
Supplement: Supplementary file 1 [file Data_Sheet_1.pdf]

Appendix A

Linear mixed effects model for three time point analysis including crossover effects.

	GABA				Glx				NAA			
	Full LME		Crossover LME		Full LME		Crossover LME		Full LME		Crossover LME	
	**B (95%CI)**	**p-value**	**B (95%CI)**	**p-value**	**B (95%CI)**	**p-value**	**B (95%CI)**	**p-value**	**B (95%CI)**	**p-value**	**B (95%CI)**	**p-value**
Intercept	3.16 (2.93,3.38)	<0.001	3.21 (2.98,3.44)	<0.001	8.1 (7.53,8.68)	<0.001	8.22 (7.63,8.81)	<0.001	10.47 (10.13,10.82)	<0.001	10.77 (10.43,11.1)	<0.001
Time	-	0.756	-	0.562	-	0.329	-	0.143	-	0.376	-	0.556
dur1 vs. pre1	-0.06 (-0.32,0.21)	-	-0.11 (-0.3,0.07)	-	0.01 (-0.67,0.7)	-	0.06 (-0.43,0.55)	-	0.18 (-0.17,0.53)	-	-0.04 (-0.29,0.21)	-
post1 vs. pre1	-0.14 (-0.41,0.12)	-	-0.12 (-0.3,0.07)	-	0.53 (-0.16,1.21)	-	0.35 (-0.14,0.84)	-	0.17 (-0.18,0.51)	-	-0.01 (-0.26,0.25)	-
pre2 vs. pre1	-0.08 (-0.4,0.25)	-	-	-	-0.35 (-1.16,0.46)	-	-	-	0.5 (0.01,0.98)	-	-	-
dur2 vs. pre1	-0.25 (-0.57,0.08)	-	-	-	-0.25 (-1.06,0.57)	-	-	-	0.23 (-0.25,0.72)	-	-	-
post2 vs. pre1	-0.16 (-0.49,0.16)	-	-	-	-0.18 (-0.99,0.64)	-	-	-	0.32 (-0.17,0.8)	-	-	-
Cross over effect	-	-	-0.06 (-0.17,0.05)	0.671	-	-	-0.19 (-0.48,0.09)	0.902	-	-	-0.03 (-0.18,0.12)	0.813
Group effect	-0.15 (-0.47,0.18)	0.983	-0.15 (-0.34,0.03)	0.132	-0.02 (-0.83,0.8)	0.224	0.13 (-0.36,0.62)	0.764	0.47 (-0.02,0.95)	0.336	0.13 (-0.12,0.38)	0.374
Change between groups over time	-	0.92	-	0.262	-	0.527	-	0.172	-	0.428	-	0.679
dur1 vs. pre1	0 (-0.37,0.37)	-	0.11 (-0.16,0.37)	-	-0.05 (-1.02,0.92)	-	-0.15 (-0.85,0.54)	-	-0.34 (-0.83,0.14)	-	-0.11 (-0.47,0.24)	-
post1 vs. pre1	0.1 (-0.27,0.47)	-	0.1 (-0.16,0.36)	-	-0.5 (-1.46,0.47)	-	-0.11 (-0.8,0.58)	-	-0.08 (-0.56,0.41)	-	-0.08 (-0.44,0.28)	-
pre2 vs. pre1	-0.01 (-0.54,0.52)	-	-	-	0.3 (-1.01,1.6)	-	-	-	-0.68 (-1.51,0.16)	-	-	-
dur2 vs. pre1	0.21 (-0.32,0.74)	-	-	-	0.04 (-1.27,1.35)	-	-	-	-0.56 (-1.39,0.28)	-	-	-
post2 vs. pre1	0.09 (-0.44,0.63)	-	-	-	0.57 (-0.73,1.88)	-	-	-	-0.76 (-1.6,0.07)	-	-	-

Pre = pre-stimulation window, dur = during stimulation window, post = post-stimulation window. Numbers denote session 1 = first, 2 = second. LME = linear mixed effects model. 95% CI = 95% confidence interval.

Appendix B

Linear mixed effects model for five time point analysis including crossover effects.

	GABA				Glx				NAA			
	Full LME		Crossover LME		Full LME		Crossover LME		Full LME		Crossover LME	
	B (95%CI)	p-value	B (95%CI)	p-value	B (95%CI)	p-value	B (95%CI)	p-value	B (95%CI)	p-value	B (95%CI)	p-value
Intercept	3.16 (2.91,3.4)	<0.001	3.21 (2.99,3.43)	<0.001	8.1 (7.49,8.72)	<0.001	8.18 (7.6,8.76)	<0.001	10.47 (10.11,10.84)	<0.001	10.75 (10.43,11.08)	<0.001
Time	-	0.826	-	0.808	-	0.124	-	0.176	-	0.606	-	0.582
dur1-1 vs. pre1	-0.13 (-0.41,0.15)	-	-0.11 (-0.31,0.09)	-	0.09 (-0.67,0.85)	-	-0.02 (-0.56,0.53)	-	0.12 (-0.26,0.49)	-	-0.07 (-0.34,0.2)	-
dur1-2 vs. pre1	-0.02 (-0.3,0.26)	-	-0.09 (-0.29,0.11)	-	-0.1 (-0.86,0.67)	-	0.03 (-0.52,0.57)	-	0.11 (-0.27,0.48)	-	-0.03 (-0.3,0.24)	-
post1-1 vs. pre1	-0.13 (-0.41,0.15)	-	-0.08 (-0.29,0.12)	-	0.77 (0.01,1.53)	-	0.41 (-0.14,0.96)	-	0.03 (-0.34,0.4)	-	-0.09 (-0.36,0.18)	-
post1-2 vs. pre1	-0.1 (-0.38,0.19)	-	-0.14 (-0.34,0.06)	-	0.21 (-0.56,0.97)	-	0.29 (-0.26,0.84)	-	0.15 (-0.22,0.53)	-	0.04 (-0.22,0.31)	-
pre2 vs. pre1	-0.08 (-0.42,0.27)	-	-	-	-0.35 (-1.22,0.52)	-	-	-	0.5 (-0.02,1.02)	-	-	-
dur2-1 vs. pre1	-0.17 (-0.51,0.18)	-	-	-	-0.47 (-1.34,0.4)	-	-	-	0.24 (-0.28,0.76)	-	-	-
dur2-2 vs. pre1	-0.24 (-0.58,0.11)	-	-	-	-0.2 (-1.07,0.66)	-	-	-	0.33 (-0.19,0.85)	-	-	-
post2-1 vs. pre1	-0.12 (-0.47,0.23)	-	-	-	-0.3 (-1.17,0.56)	-	-	-	0.28 (-0.24,0.8)	-	-	-
post2-2 vs. pre1	-0.26 (-0.6,0.09)	-	-	-	0.03 (-0.84,0.9)	-	-	-	0.43 (-0.08,0.95)	-	-	-
Cross over effect	-	-	-0.06 (-0.15,0.03)	0.961	-	-	-0.17 (-0.41,0.08)	0.989	-	-	-0.02 (-0.14,0.1)	0.404
Group effect	-0.15 (-0.5,0.2)	0.999	-0.15 (-0.35,0.05)	0.038	-0.02 (-0.88,0.85)	0.095	0.13 (-0.42,0.68)	0.516	0.47 (-0.05,0.99)	0.51	0.13 (-0.14,0.4)	0.821
Change between groups over time	-	0.99	-	0.158	-	0.504	-	0.175	-	0.541	-	0.737
dur1-1 vs. pre1	0.07 (-0.33,0.46)	-	0.09 (-0.2,0.37)	-	-0.07 (-1.14,1.01)	-	-0.07 (-0.84,0.71)	-	-0.17 (-0.7,0.35)	-	-0.05 (-0.44,0.33)	-
dur1-2 vs. pre1	-0.08 (-0.48,0.32)	-	0.05 (-0.24,0.33)	-	-0.06 (-1.13,1.02)	-	-0.02 (-0.8,0.76)	-	-0.46 (-0.99,0.07)	-	-0.27 (-0.65,0.11)	-
post1-1 vs. pre1	0.04 (-0.36,0.44)	-	0.05 (-0.24,0.33)	-	-0.55 (-1.63,0.52)	-	-0.18 (-0.96,0.6)	-	0.12 (-0.4,0.65)	-	0 (-0.38,0.39)	-
post1-2 vs. pre1	0.08 (-0.32,0.48)	-	0.1 (-0.18,0.39)	-	-0.26 (-1.33,0.82)	-	0.02 (-0.76,0.79)	-	-0.28 (-0.81,0.25)	-	-0.26 (-0.64,0.12)	-
pre2 vs. pre1	-0.01 (-0.58,0.57)	-	-	-	0.3 (-1.06,1.66)	-	-	-	-0.68 (-1.57,0.22)	-	-	-
dur2-1 vs. pre1	0.1 (-0.47,0.67)	-	-	-	0.23 (-1.13,1.59)	-	-	-	-0.61 (-1.51,0.28)	-	-	-
dur2-2 vs. pre1	0.16 (-0.41,0.74)	-	-	-	0.31 (-1.05,1.67)	-	-	-	-0.76 (-1.65,0.14)	-	-	-
post2-1 vs. pre1	0.05 (-0.53,0.62)	-	-	-	0.49 (-0.87,1.85)	-	-	-	-0.79 (-1.68,0.1)	-	-	-
post2-2 vs. pre1	0.11 (-0.46,0.69)	-	-	-	0.59 (-0.77,1.95)	-	-	-	-0.91 (-1.81,-0.02)	-	-	-

Pre = pre-stimulation window, dur = during stimulation window, post = post-stimulation window. Numbers x-y denote session and window number, where x = denotes the session and y-denotes the window. LME = linear mixed effects model. 95% CI = 95% confidence interval.
